# Estimating the Availability of Potential Homes for Unwanted Horses in the United States

**DOI:** 10.3390/ani7070053

**Published:** 2017-07-20

**Authors:** Emily Weiss, Emily D. Dolan, Heather Mohan-Gibbons, Shannon Gramann, Margaret R. Slater

**Affiliations:** Research and Development, Community Outreach, American Society for the Prevention of Cruelty to Animals (ASPCA ^®^), New York, NY 10128, USA; emily.dolan@aspca.org (E.D.D.); heather.mohan-gibbons@aspca.org (H.M.-G.); shannon.gramann@aspca.org (S.G.); margaret.slater@aspca.org (M.R.S.)

**Keywords:** horses, slaughter, rescue, adoption

## Abstract

**Simple Summary:**

There are approximately 200,000 unwanted horses annually in the United States. Many are shipped to slaughter, enter rescue facilities, or are held on federal lands. This study aimed to estimate a potential number of available homes for unwanted horses in order to examine broadly the viability of pursuing re-homing policies as an option for the thousands of unwanted horses in the U.S. The results of this survey suggest there could be an estimated 1.2 million homes who have both the perceived resources and desire to house an unwanted horse. This number exceeds the approximately 200,000 unwanted horses living each year in the United States. These data suggest that efforts to reduce unwanted horses could involve matching such horses with adoptive homes and enhancing opportunities to keep horses in the homes they already have.

**Abstract:**

There are approximately 200,000 unwanted horses annually in the United States. This study aimed to better understand the potential homes for horses that need to be re-homed. Using an independent survey company through an Omnibus telephone (land and cell) survey, we interviewed a nationally projectable sample of 3036 adults (using both landline and cellular phone numbers) to learn of their interest and capacity to adopt a horse. Potential adopters with interest in horses with medical and/or behavioral problems and self-assessed perceived capacity to adopt, constituted 0.92% of the total sample. Extrapolating the results of this survey using U.S. Census data, suggests there could be an estimated 1.25 million households who have both the self-reported and perceived resources and desire to house an unwanted horse. This number exceeds the estimated number of unwanted horses living each year in the United States. This study points to opportunities and need to increase communication and support between individuals and organizations that have unwanted horses to facilitate re-homing with people in their community willing to adopt them.

## 1. Introduction

Estimates of the total number of horses in the U.S. vary widely but the number most often cited, 9.2 M, comes from a 2005 economic impact study commissioned by the American Horse Council (AHC) [1]. There are many thousands of unwanted horses annually in the United States. Unwanted is defined by the Unwanted Horse Coalition [1] (a program of the AHC) as “horses which are no longer wanted by their current owner because they are old, injured, sick, unmanageable, fail to meet their owner’s expectations (e.g., performance, color or breeding), or their owner can no longer afford them”. The reasons horses are unwanted are varied. An estimated 6000–10,000 horses are housed in horse rescues at any given time [2,3]. One U.S. study found that the most common reasons horses were relinquished to rescue organizations were health (54%), lack of suitability for desired purpose (28%), and behavioral problems of the horses (28%) [2]. Owner-related factors most commonly reported were financial hardship (52%) physical illness or death of the owner (27%), and lack of time for the horse (16%) [2]. Horses who were relinquished were most commonly thoroughbreds (22%) and quarter horses (19%) and 51% were geldings, 7.5% colts/stallions and 42% mares. A wide range of ages were reported with a mean of 12 years old. In another national U.S. study of horses seized in cruelty, neglect or abandonment investigations, the most common reasons leading to the investigation were: owner ignorance, economic hardship, and lack of responsibility [4]. Many unwanted, but otherwise re-homable, horses are among the estimated 82,000 to 150,000 horses that are shipped annually to Mexico or Canada for slaughter [5,6,7,8]. Among the horses shipped to slaughter between 2002 and 2005, the demographics were similar to the U.S. horse population, indicating that the option of slaughter was applied across the spectrum of horse ownership [9]. There are also more than 100,000 horses being held long-term on open lands by the Bureau of Land Management (both on and off-range) [10].

A number of options exist for unwanted horses, including relinquishment to rescue organizations, donation to universities or law enforcement agencies, sale to or adoption by new owners (re-homed) or euthanasia [9]. For some of these outcomes, the horses must meet specific criteria; for others, the owner must be aware of the option, there must be space available in the program for the horse, or the owner must be able to afford euthanasia and disposal. Increasing the ability of existing horse owners to re-home their horses to private households is one potential way to reduce the number of unwanted horses and improve their welfare and longevity. Horses typically have multiple owners [11], can live up to 30 years [7,11], and are expensive to keep [12,13] making life-long housing difficult to ensure. In order to determine if re-homing is a viable option, it is important to know if there are enough homes to accommodate the number of unwanted horses. To our knowledge, there is no current evidence to inform this question.

This study used a national survey to gather information about the number of potential homes for these horses in a “horse-interested population” (defined as currently owning a horse, having owned a horse in the past 5 years, or interested in owning a horse in the near future). This survey examined whether people would be willing to adopt unwanted horses, what characteristics were required of horses to be considered “adoptable” in the respondent’s opinion, and whether potential adopters thought they had adequate resources to keep a horse. From this survey, an estimate for the number of potential homes for horses in the United States was extrapolated in order to broadly examine the viability of pursuing re-homing policies as an option for the thousands of unwanted horses in the U.S.

## 2. Materials and Methods

### 2.1. Survey

A telephone-based survey of the general adult population was conducted by Edge Research using CARAVAN ^®^ ORC International. Telephone calls were made between 24 September 2015 and 11 October 2015. The CARAVAN ^®^ Omnibus telephone survey is a nationally projectable study conducted among a probability sample of U.S. residents, 18 years of age and older. See Appendix A for ORC International’s complete methodology. The horse survey questions were included in a larger bank of questions asked during the interview. This study was conducted using two probability samples: randomly selected landline telephone numbers and randomly selected mobile (cellular) telephone numbers. The combined sample consists of 3036 adults (18 years old and older) living in the continental United States. Of the 3036 interviews, 1536 were from the landline sample and 1500 from the cell phone sample. The survey had a response rate of 20% and a completion rate of 86%.

The survey sample size was selected to serve two purposes. First, we wanted to ensure that the estimate of potential adoptive households for horses in the U.S. (the primary outcome) was sufficiently precise (i.e., had a narrow enough confidence interval) to be useful in practice. For hypothetical scenarios where either 0.5%, 1.0%, or 1.5% of households were interested in and capable of adopting a horse, an overall sample size of 3000 would ensure confidence interval coverage of no more than ±0.5%. In other words, the 95% confidence interval would cover less than 0.5% in either direction from the point estimate for all three scenarios. Secondly, we continued the survey to ensure that at least 500 respondents were *horse-interested*. This ensured that the confidence interval coverage would be no more than ±5% for any proportions calculated from this subgroup.

### 2.2. Survey Questions

To estimate the number of individuals with available homes, respondents were asked: “Which, if any, types of animals do you personally own?”; “Have you ever owned a horse?”; “How long ago did you last own a horse?”; “How interested are you in obtaining a horse at some point in the future?” with possible responses “very interested”, “somewhat interested”, and “not interested”; and for three scenarios (1. A horse that no longer has an owner; 2. A horse that has medical or behavioral challenges; 3. A horse that might be abandoned if a new owner is not found): “…how interested you would be in adopting a horse in those circumstances” with possible responses “very interested”, somewhat interested”, and “not interested”; and “Do you currently have the space and resources necessary to house and care for a newly-adopted horse on your own property or at a local barn or stabling facility?” with possible responses “yes”, “no”, or “don’t know”.

### 2.3. Final Sample

Among the total sample of individuals reached, we first identified those who were *horse-interested* as defined by reporting currently having or having had a horse (in the last 5 years) and/or being interested in getting a horse in the future. Among these, we further defined a target subgroup of *potential adopters* who have the interest in and perceived resources and capacity to take a re-homed horse based on two additional criteria. To qualify for this subgroup, respondents must first have reported *strong interest* in adoption under all 3 scenarios of interest (a horse facing abandonment, a horse without an owner, and a horse with medical or behavioral problems). We used the criterion of strong interest to ensure that casual or circumstantial interest was not included in the definition. Secondly, the respondent must also have endorsed currently perceiving that they have the space and resources to care for a horse. A further, smaller, subgroup was *experienced potential adopters*, which included only those *potential adopters* who also have previous experience owning a horse in the last 5 years, indicating that they had an understanding of the demands needed to care for a horse.

### 2.4. Analysis

Characteristics of respondents were described using frequencies and percentages. We used proportions from our sample to make inferences about the corresponding population level proportions using standard statistical methods [14]. For key results, exact 95% confidence intervals were calculated using the standard Cloppe-Pearson method in our software [15]. The confidence intervals generated can be interpreted as a plausible range for the proportion of the U.S. population that would provide the same answers. This method was selected because it provides more conservative estimates (i.e., wider confidence intervals) for small proportions (i.e., accounts for uncertainty in the population level estimates that could be related to relying on a random sample). Numbers of U.S. households and U.S. adults meeting our definitions of *horse-interested* and *potential adopters* were estimated by multiplying proportion estimates and lower and upper confidence intervals with corresponding population totals [16]. Demographics of *potential adopters* were compared to other *horse-interested* respondents using the chi-square test. For adults in the U.S., 2015 total population of 321,418,820 was multiplied by the percentage of adults in the U.S. in 2015 (77.1%, with the percentage of the population <18 = 22.9%) [16]. This equaled ~247,813,910 U.S. adults. For total U.S. households, the estimated number in 2016 was 135,697,926 [16]. Analyses were run using Stata/IC 13.1 (StataCorp LP, College Station, TX, USA).

### 2.5. Ethical Statement

All respondents were informed that their responses would be kept confidential. The only identifying information collected was their first name; recorded for quality control purposes. Institutional review was not sought because the data were collected as part of a larger, national opinion survey and this type of research is considered to be exempt from review by IRBs.

## 3. Results

### 3.1. Demographics

Data describing the characteristics of the national sample and the *horse-interested* sample are in Table 1.

### 3.2. Sample Breakdown Based on Responses

**Figure 1:**
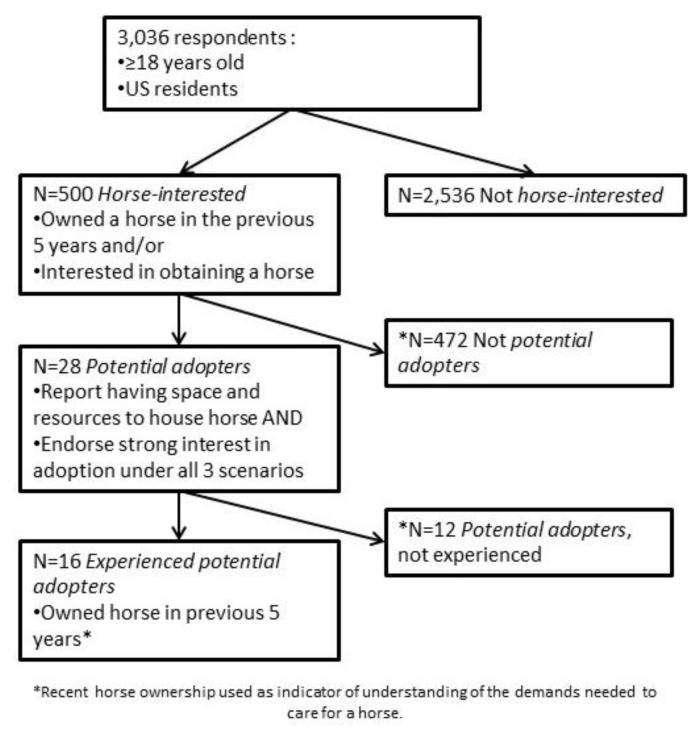
Proportion of respondents in each category.

The breakdown of respondents, in a flow diagram, is shown in Figure 1.

**Figure 2:**
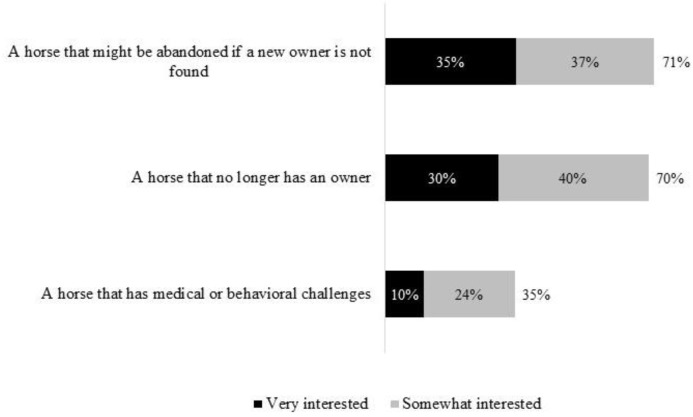
Reported interest by 500 *horse-interested* U.S. residents in adopting unwanted horses under various scenarios.

Among the 3036 individuals contacted, seventeen percent (95% CI 15–18%; *n* = 500) met our criteria for *horse-interested* by reporting that they either currently own a horse, want to own a horse in the near future, or have owned a horse within the past 5 years. These respondents then reported their interest in horse ownership based upon three common scenarios, shown in Figure 2.

Nine percent (45; 95% CI 7–12%) of the *horse-interested* sample reporting being “very interested" in obtaining a horse under all three scenarios. Further, 46% of the *horse-interested* sample (230; 95% CI 42–51%) reported having the resources (which we termed perceived capacity) to house and care for a newly-adopted horse.

Among the *horse-interested* sample, 5.6% (28; 95% CI 4–8%) were classified as *potential adopters* based on reporting both strong interest in each of the three adoption scenarios as well as having the perceived capacity to house and care for a horse. These 28 *potential adopter* respondents represent 0.92% (CI = 0.6–1.3%) of the total sample. There were no significant or important differences between *potential adopters* and other horse interested responders (gender *p* = 0.5; age *p* = 0.5; income *p* = 0.7). Among *potential adopters*, 53% were women, 32% were 18–29 years old, 36% 30–49 years old, 25% 50–64 years old and 7% age 65 and up. For income, 30% of *potential adopters*’ income was <U.S. $25,000, 26% was $25,000 to <$50,000, 29% from $50,000 to <100,000 and 26% had incomes of $100,000 and above.

### 3.3. Population Estimates

Applying the percentage of *potential adopters* to the number of U.S. households yielded an estimated 1.25 million households (0.0092 × 135,697,926; CI = 0.83–1.80 million) that had strong interest and perceived they could house a horse. Multiplying 0.92% times the U.S. population of 247,813,901 adults for a less conservative estimate, we estimated that approximately 2.28 million people (CI = 1.52–3.30 million) in the U.S. would have strong interest in obtaining a horse and perceived they could house a horse.

Twelve respondents who met the criteria through their interest in future horse ownership had not previously owned a horse. Excluding those from the 28, 16 or 0.53% (CI = 0.3–0.9%) *experienced potential adopters* reported strong interest, perceived capacity, and currently or had ever owned a horse. Applying this more conservative criteria, an estimated 0.72 million U.S. households (CI = 0.41–1.22 million), with first-hand knowledge of the challenges of owning a horse, could house a horse.

## 4. Discussion

There are many reasons why horses may be abandoned and in need of re-homing. One central reason is affordability; however, owners reported a variety of more specific reasons including old age, injuries, horse behavior, factors in the owner’s household such as divorce or lack of time and the horse not meeting expectations [2,8]. Regardless of the underlying industry reasons for the numbers of horses needing to be re-homed, finding previously untapped homes is critical. The current study found an estimated 1.25 million households with the interest and self-assessed, perceived capacity to adopt a horse in the United States, including those with medical or behavioral problems. Accounting for the uncertainty that comes with applying a sample proportion to the entire population, the true count could reasonably be expected to lie between 0.83 and 1.80 million households. This estimate was based on only those surveyed who reported strong interest in all three categories of unwanted horses: a horse that might be abandoned; a horse that is homeless; a horse with medical or behavioral issues. Excluding qualified respondents who had never owned a horse, 0.72 million households are estimated to have the perceived capacity and interest in owning a horse. These numbers of interested households who perceive themselves as qualified, suggests that there are more available homes than previously thought to accommodate unwanted horses in the U.S.

The number of respondents in our survey was large and this probability sample was representative of the population of the United States, indicating that our estimates are reflective of national interest in horse ownership. When using a less conservative approach by estimating based on individuals rather than households, 2.28 million individuals reported self-assessed perceived capacity to house horses. We looked at individual estimates because we assumed respondents to be from different households and because each individual with interest might be engaged in horse related support programs. However, we acknowledge that it is more conservative to consider households as the basis for estimating new homes for horses.

What is not clear is how long the number of potential homes would exceed the number of horses that need those homes. It is theorized that as horses move amongst homes, the availability of new homes would likely remain stable. Further, although the lifespan of a horse is not short, each year, homes with horses have horses that die or are euthanized, opening some spaces for new horses. It could be, however, that the number of homes would eventually become saturated. If that were true, this estimate remains crucial for three primary reasons: (1) even just a short time of reducing the prevalence of unwanted horses in the United States would free available resources for expanded access to safety net services and resources in order to reduce the incidence of newly unwanted horses; (2) the estimate provides a rich and substantial target for recently launched innovative industry programs such as Time To Ride [17], which aims to grow the horse industry though engaging new audiences, and The Right Horse [18], which aims to promote horse adoption in innovative ways; and (3) moving even a small number of additional horses into homes would allow for a decreased prevalence of unwanted horses.

In addition to increasing the likelihood of finding more homes for horses, there may be opportunities to keep horses in their current homes with more community support. Given limited capacity and funding of rescue organizations [2,8], safety net and expanded programs can improve the industries’ reach. Equine rescue organizations in the U.S. have an estimated capacity of 13,400 horses per year [2]. Given that 53% of those horses arrived in poor health or body condition and that 23% of owners reported economic or financial hardship as a contributor to relinquishment [2], programs which better support current owners and their horses with food and/or accessible and affordable veterinary care have promise in potentially preventing re-homing. Another survey of horse owners [19] found that 47% noted they believed cost of care was a problem and a contributing factor to unwanted horses. It is possible that providing assistance to horse owners in times of crisis could keep some horses in their current homes, preventing the re-homing in the first place. These shifts, as well as support for end-of-life, including providing affordable, accessible, humane euthanasia [20], would allow resources to focus on moving horses from conditions of neglect and cruelty toward higher welfare living arrangements.

It is possible that some of those people who noted having both strong interest and perceived capacity would not actually adopt when given the opportunity. Further, the present study only considered the respondents perceptions and self-assessment of whether they have adequate resources to care for a horse and not any objective measures of the adequacy of the available resources. It is acknowledged that reported intent could overestimate actual behavior. However, the responders’ ability to house and care for a horse may or may not be related to previous horse ownership. Previous owners may know what is needed to appropriately house a horse or may continue to provide care which some might view as less than optimal. New horse owners could spend time becoming well informed or simply dive into horse ownership. We strongly encourage additional information sharing with current and new horse owners as an additional method of improving horse welfare. The data presented, however, suggest preliminarily that there may be many homes for unwanted horses that have not yet been accessed. Surprisingly, people showed interest in adopting horses that needed extra support, as 35% of respondents reported being “very or somewhat interested” in adopting a horse that was medically or behaviorally unsound. It is possible that some responses were due to social desirability bias, where the respondents gave the socially correct or pleasing answer. That would tend to increase the likelihood of respondents saying that would be interested in adopting a horse. In this survey, the presence of multiple other questions and the neutral organization administering the survey, as well as the lack of face to face interaction would partially help to mitigate this bias [21,22].

Because the characteristics of horses likely contribute to their ability to be re-homed [23], research into increasing access to services such as medical, and behavior support would provide important information in expanding the link between unwanted horses and potential adopters. This would also be important to ensure that adopters of horses with medical or behavior issues receive the counseling and support needed to appropriately care for those horses.

The current results that there are currently untapped potential adopters seems to contradict previously published results that shelters and rescues are overwhelmed [2]. In general, this contradiction points to a potential gap in communication or understanding between horse organizations that have horses available for adoption and people interested in adopting. It is possible that horse organizations have not embraced the importance of finding new homes as an important part of allowing them to save more horses. Additionally, finding new and creative ways to connect potential homes with current owners who need to re-home is likely to be critically important [22]. Future research that focuses on the opportunities to increase adoptions from horse organizations would provide valuable information to the body of unwanted horse literature. A study of people who have re-homed their horses is currently being conducted to complement the findings presented here and to update findings in Holcomb et al. [20].

## 5. Conclusions

This study found an estimated 1.24 million households interested in and potentially able to adopt a horse and 2.26 million potential horse advocates for horses in need. While this estimate may not reflect an immediate, objectively suitable set of adopters, these numbers of people who report their willingness and perceived ability exceed the known estimates of unwanted horses and suggest a substantial and underutilized resource. This study points to opportunities to increase communication and support between individuals and organizations that have unwanted horses to facilitate re-homing with people in their community willing to adopt them.

## Figures and Tables

**Table 1:** The unweighted frequencies and percentages for the nationally representative sample of 3036 respondents and the 500 who were *horse-interested*.

Respondent Characteristics	National Sample, *n* = 3036		*Horse-Interested* Sample, *n* = 500	
	Frequency	Percent	Frequency	Percent
Gender				
Male	1529	50.4	262	52.4
Female	1507	49.6	238	47.6
Total	3036	100.0	500	100.0
Age range				
18–29	473	15.5	126	25.2
30–49	678	22.3	148	29.6
50–64	888	29.3	146	29.2
65 or older	961	31.7	77	15.4
Refused/No Response	36	1.2	3	0.6
Total	3036	100.0	500	100.0
Total household income (before tax, 2014)				
Under U.S.$25,000	585	19.3	107	21.4
$25,000 but less than $50,000	735	24.2	144	28.8
$50,000 but less than $100,000	628	20.7	91	18.2
$100,000 or more	519	17.1	80	16.0
Don‘t know/Refused/NR	569	18.7	78	15.6
Total	3036	100.0	500	100.0
Region				
North East	551	18.2	75	15.0
Midwest	675	22.2	97	19.4
South	1136	37.4	208	41.6
West	674	22.2	120	24.0
Total	3036	100.0	500	100.0

## Appendix A

ORC International’s Random Digit Dial (RDD) telephone sample is generated using a list-assisted methodology. The standard GENESYS RDD methodology produces a strict single stage, EPSEM (Equal Opportunity of Selection Method) sample of residential telephone numbers. The cell phone sample, also RDD, has been supplied by SSI, Inc. using their proprietary Cell/WINS technology. The cell phone sample is generated from cell phone 1000 series blocks (a “block” is defined by the first seven digits of the phone number) with all permutations within each block included. The sampling interval is then calculated by dividing the universe of all possible numbers by the number of records desired, thus specifying the size of the frame subdivisions. Within each of the subsets, one number is selected at random giving all numbers an equal probability of selection.

Surveys are collected by trained and supervised U.S. based interviewers using ORC International’s computer assisted telephone interviewing (CATI) system. Final data are adjusted to consider the two sample frames and then weighted by age, gender, region, race/ethnicity and education to be proportionally representative of the U.S. adult population. Weighting adjustments are used to reduce the potential for biases that may be present due to incomplete frame coverage and survey nonresponse-both inherent in all telephone surveys. Each respondent is given a score based on their reported information and then either weighted up (if they are underrepresented) or weighted down (if they are overrepresented).
